# Impact of early high protein intake in critically ill patients: a randomized controlled trial

**DOI:** 10.1186/s12986-024-00818-8

**Published:** 2024-06-28

**Authors:** Yifei Wang, Yanyang Ye, Lusha Xuan, Lijie Xu, Pengpeng Wang, Jun Ma, Yuyan Wang, Yanjun Chen, Jinli Miao, Wenmin Wang, Lingjie Zhou

**Affiliations:** 1 Intensive Care Unit, Zhuji Traditional Chinese Medical Hospital, Zhuji, 311800 Zhejiang China; 2 Department of science and education, Zhuji Traditional Chinese Medical Hospital, Zhuji, 311800 Zhejiang China; 3 The Yangtze River Delta Biological Medicine Research and Development Center of Zhejiang Province, Yangtze Delta Region Institution of Tsinghua University, Hangzhou, 314006 Zhejiang China

**Keywords:** Critically ill patients, Early high protein intake, Enteral nutrition, Prognosis, Septic patients

## Abstract

**Background:**

Conflicting findings regarding the impact of High protein intake during the early phase in critically ill patients have been reported. Therefore, we aimed to assess the influence of higher early protein intake on the prognosis of critically ill patients.

**Methods:**

This randomized controlled trial involved 173 critically ill patients who stayed in the Intensive Care Unit/Emergency ICU (ICU/EICU) for at least 7 days. The Low group (n = 87) and High group (n = 86) received protein supplementation of 0.8 g/kg.d and 1.5 g/kg.d, respectively, within 1–3 days of enteral nutrition (EN) initiation, with both groups transitioning to 1.5 g/kg.d on the 4th day. The serum prealbumin (PA), blood urea nitrogen/creatinine, and rectus femoris muscle thickness and cross-sectional area of all patients was measured on the 1th, 3rd, 5th, 7th day, and the day of ICU/EICU discharge.

**Results:**

Patients in both Low and High groups showed no significant differences in age, APACHE II scores, or other demographic and baseline characteristics. There were also no significant differences in the primary outcome (28-day mortality rate) and secondary outcomes (incidence rate of refeeding syndrome and EN tolerance score) between the two groups. However, the Low group exhibited a significantly higher 28-day mortality rate (HR = 2.462, 95% CI: 1.021–5.936, P = 0.045) compared to High group, as determined by Cox proportional hazards models incorporating the time factor. The High group exhibited significantly shorter durations of mechanical ventilation and ICU stay compared to the Low group. Serum PA levels were higher, and rectus femoris muscle atrophy rates were lower in the High group. Furthermore, for septic patients, high protein intake significantly reduced the 28-day mortality rate despite a small sample size (n = 34).

**Conclusions:**

Our study indicates that increasing early protein intake to 1.5 g/kg.d may be safe and help improve the nutritional status and prognosis of critically ill patients.

**Trial registration:**

This study was registered with the Chinese Clinical Trial Registry (ChiCTR2000039997, https://www.chictr.org.cn/).

**Supplementary Information:**

The online version contains supplementary material available at 10.1186/s12986-024-00818-8.

## Introduction

Nutrition intake and management play a crucial role in the treatment and recovery of diseases, with particular significance in the Intensive Care Unit (ICU) and Emergency ICU (EICU). Nutritional therapy for critically ill patients has emerged as a research hotspot in recent years. This therapeutic approach aims to provide adequate energy and substrate for cellular metabolism, maintain tissue and organ structure and function, correct metabolic disorders, regulate immunity, and improve existing or potential nutritional deficiencies. These strategies collectively aim to influence disease progression and improve patient outcomes [1]. While most studies assessing the impact of nutrition support on ICU clinical outcomes have focused on energy supply [2, 3], an increasing amount of evidence suggests that protein intake may be more critical than calorie intake [4, 5]. Critically ill patients experience significantly greater protein breakdown metabolism than synthesis, especially in the early stages. This imbalance is closely associated not only with ICU-acquired muscle weakness but also with immunosuppression, impaired wound healing, and other adverse consequences [6, 7]. Therefore, appropriate protein provision can meet metabolic demands without increasing metabolic burden, offering benefits to patients.

However, controversy persists regarding the optimal dose and timing of protein supplementation in the early stages of disease. Both the American Society for Parenteral and Enteral Nutrition (ASPEN) and the European Society for Clinical Nutrition and Metabolism (ESPEN) recommend that critically ill patients should receive more protein than healthy individuals. For critically ill patients with a BMI < 30, protein requirements should be in the range of 1.2–2.0 g/kg.d, with potentially higher amounts needed for burn and trauma patients [1, 6]. Additionally, for critically ill patients requiring nutritional support, enteral nutrition (EN) is preferred over parenteral nutrition (PN) [1]. However, in the early stages of critical illness, significant stress and alterations in neuroendocrine factors result in the body relying on glycogen, endogenous protein breakdown, and fat mobilization for energy, with reduced external energy requirements [8]. Excessive protein intake during this period may not be absorbed and lead to adverse reactions such as azotemia and hyperglycemia, thereby increasing the burden on the liver and kidneys and adversely affecting clinical outcomes [9]. Koekkoek et al. found a time-dependent relationship between protein intake and mortality in mechanically ventilated ICU patients. Higher protein intake in the first 3 days of ICU admission was associated with increased mortality, whereas higher protein intake beyond 3 days was associated with lower mortality [4].

In a previous observation of 20 ICU patients, early high protein supplementation did not improve nutritional status but increased the incidence of gastrointestinal intolerance, with no impact on 28-day mortality. In light of these findings and in accordance with clinical guidelines of CONSORT [1, 6], we conducted a prospective randomized controlled study building upon preliminary research. The aim of this study was to investigate the impact of early high protein intake on critically ill patients and to determine the effective and safe dose. We assessed the effects of low protein intake (0.8 g/kg.d) and high protein intake (1.5 g/kg.d) through EN on clinical outcomes, nutritional biochemical indicators, and rectus femoris atrophy rates in critically ill patients.

## Methods

### Study design and population

This study is a prospective, single-blind, randomized controlled trial with two parallel treatment arms conducted at the Chinese Medicine Hospital in Zhuji City (Zhejiang, China). Ethics Committee of Zhuji Traditional Chinese Medicine Hospital (NO.2020-KYSB-005) gave approval for this study. Informed consent was obtained from all patients’ legal guardians before their inclusion in the study, and they signed the clinical research informed consent form. This study was registered with the Chinese Clinical Trial Registry (ChiCTR2000039997, https://www.chictr.org.cn/).

**Fig. 1:**
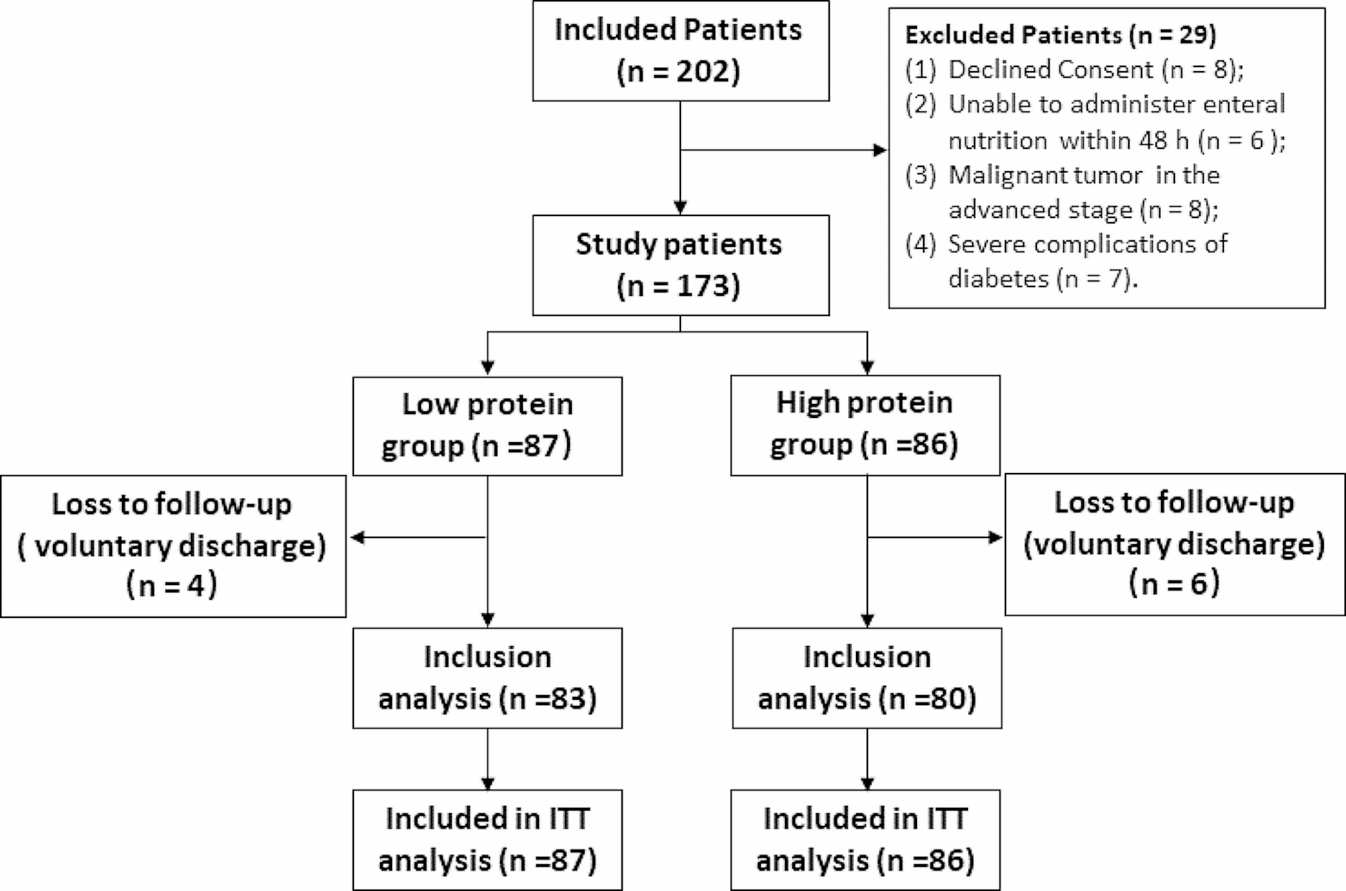
Flow chart

Patient recruitment occurred from January 1, 2021, to April 30, 2023. Eligibility criteria included critically ill patients aged above 18 years admitted to ICU and EICU. Patients with a modified Nutrition Risk in Critically Ill (mNUTRIC) score > 5 and an anticipated ICU stay ICU/EICU of more than 7 days were included. Details of the mNUTRIC scoring criteria are presented in Supplementary Table 1. Patients with the following characteristics were excluded from the study: (1) burns; (2) inability to initiate EN within 48 h of ICU admission; (3) initiation of renal replacement therapy within 24–48 h of ICU/EICU admission; (4) pregnancy or lactation; (5) advanced malignant tumors; (6) severe complications of diabetes such as ketoacidosis, hyperosmolar coma, or acidosis; (7) a history of digestive tract surgery, primary gastrointestinal injury or neuromuscular diseases (affecting protein absorption or metabolism); (8) body mass index (BMI) ≥ 30 kg/m^2^; (9) hyperthyroidism; (10) unwillingness to participate in the study. A total of 202 patients underwent eligibility screening, with 173 participating in the study. Four patients in the Low group and six in the High group self-discharged, leading to loss to follow-up (Fig. 1).

### Sample size calculation

We used the formula

$${n}_{1}={n}_{2}=\frac{1}{2}{\left(\frac{{\mu }_{\alpha }+{\mu }_{\beta }}{{{sin}}^{-1}\sqrt{{P}_{1}}-{{sin}}^{-1}\sqrt{{P}_{2}}}\right)}^{2}$$

and the online sample size calculator tool (http://powerandsamplesize.com/) for sample size estimation. It has been reported that the mortality rate among critically ill adult patients is approximately 54.2% (P_1_=0.542) [10]. We hypothesize that increasing early protein intake will reduce the mortality rate to 30% (P_2_ =0.3). We set the Type I error rate (α) at 0.05 (95% confidence level) and the Type II error rate (β) at 0.2 (80% power). The final calculation indicates that approximately 64 participants are required per group. Considering potential dropout rates, we plan to recruit a total of 160 individuals, with 80 per group, for this study.

### Nutritional interventions

After inclusion, 173 subjects were randomly assigned to either the low protein intake group (Low group) or high protein intake group (High group). By utilizing a random number sequence generated through the RANDOM website (https://www.random.org/), participants were randomly assigned in a 1:1 ratio to the Low group (n = 87) and the High group (n = 86). The intervention started 1–2 days after patient admission. Both groups received EN. In the Low group, EN provided 0.8 g/kg.d of protein (ideal body weight) and 20 kcal/kg.d of non-protein calories for the first 3 days. In the High group, EN provided 1.5 g/kg.d of protein and 20 kcal/kg.d of non-protein calories for the first 3 days. From the 4th day, both groups received 1.5 g/kg.d of protein and 25 kcal/kg.d of non-protein calories. Ideal body weight (kg) = height (cm) − 100.

All subjects received active treatment for the primary disease, organ function support, and necessary antibiotic therapy. For critically ill patients presenting with hypoalbuminemia (serum albumin concentration < 30 g/L), albumin (ALB) was supplemented (20% human ALB) to maintain colloid osmotic pressure balance and uphold the physiological homeostasis of the organism. The sedation regimen was based on the PADIS (Pain, Agitation/Sedation, Delirium, Immobility, and Sleep Disruption) guidelines. For each patient, individualized pain and sedation targets were established, prioritizing pain management and adopting a strategy of light sedation. Physical activities were managed by rehabilitation therapists, who developed and executed intensive rehabilitation treatment plans tailored to the different disease stages of the patients. EN was initiated under the following conditions: (1) stable hemodynamics within 24–48 h of ICU admission, with mean arterial pressure (MAP) > 65 mmHg, lactate (Lac) < 4 mmol/L, norepinephrine (NE) < 0.2 μg/min/kg; (2) absence of contraindications to EN (gastrointestinal bleeding, perforation, intestinal obstruction, intra-abdominal pressure ≥ 25mmHg).

The initial feeding rate was set at 10–15 mL/h, with EN tolerance assessed every 4 h. Supplementary Table 2 outlines the criteria for EN tolerance. If well-tolerated, the rate was increased by 10–25 mL/h every 4–8 h. If intolerance occurred, the rate was maintained after symptomatic treatment; if intolerance persisted, the rate was halved, and so forth. If the target protein intake of 60% was not achieved after 7 days, supplemental parenteral nutrition (SPN) was added, and the patient exited the study. In case of severe EN intolerance leading to a significant deterioration of the condition, the researcher truthfully completed the adverse event record, and the patient was withdrawn from the study, treated as “lost to follow-up”, and excluded from the analysis.

### Primary outcome and secondary outcomes

The primary outcome is 28-day mortality. Secondary outcomes are (1) mechanical ventilation duration; (2) ICU discharge; (3) refeeding syndrome incidence rate; (4) enteral nutrition tolerance score; (5) liver function biochemical indicators: serum levels of prealbumin (PA); (6) renal function biochemical indicators: urea nitrogen/creatinine (BUN/Cr); (7) rectus femoris muscle thickness (RF-MLT); (8) rectus femoris cross-sectional area (RF-CSA). In this study, patients with blood phosphorus levels < 0.65 mmol/L or a decrease > 0.16 mmol/L were defined as having experienced refeeding syndrome.

### Data collection

Demographic data and baseline characteristics (including age, gender, APACHE II score, weight, BMI, sepsis, and the reason for ICU/EICU admission) of all patients will be collected before the intervention treatment. Measurements of primary and secondary outcomes will be recorded by professionals blinded to group assignment. All original data will be collected in the case records.

### Biochemical markers

The serum PA levels and urea nitrogen/creatinine (BUN/Cr) of all subjects were measured by automatic biochemical analyzer at different time points after entering ICU for treatment.

### Ultrasonography of the rectus femoris

Using Mindray-M7T color Doppler ultrasound, measurements of right thigh RF-MLT and RF-CSA were taken on the 1th, 5th day, and the day of ICU discharge. Atrophy rates of RF-MLT and RF-CSA were calculated using the formulas (D_5_-D_1_)/D_1_ and (D_x_-D_1_)/D_1_, respectively. Measurements were conducted with patients in a supine position, head elevated by 30°, and both lower limbs in a naturally relaxed and extended state. The linear probe with a frequency of 6–13 MHz was placed vertically at the mid-lower third of the right thigh between the anterior superior iliac spine and the upper edge of the patella, adjusting the probe position to display the rectus femoris muscle. Muscle thickness was measured as the vertical distance within the high echogenic fascia, and the cross-sectional area was outlined along the fascia. Each measurement was independently performed by two healthcare personnel, and the average was used for statistical analysis.

### Statistical analysis

The outcome analysis was conducted based on intention-to-treat (ITT), including all participants who were randomized into the trial. We further analyzed the 28-day mortality rate and the incidence of refeeding syndrome by incorporating the time factor using Cox proportional hazards models. We addressed the issue of missing data by applying multiple imputation with the random forest method, utilizing the ‘mice’ package in R (version 4.3.0) [11]. Continuous data were expressed as medians [interquartile ranges (IQR)] or means ± standard deviation (mean ± SD). If any group in the between-group comparison does not conform to a normal distribution, the continuous variables for both groups should be presented using medians (IQR). Categorical data were presented as numbers (%). IBM SPSS Statistics 22 (IBM, USA) and Prism 9 (GraphPad Software, USA) were used for data analysis and visualization. Independent sample T-tests or Mann-Whitney U tests were applied for continuous data, depending on normal distribution and homogeneity of variance. Differences between the two groups for categorical variables were analyzed using the chi-square test. All statistical tests were two-tailed, with a two-sided significance level of *P* < 0.05.

## Results

### Patient characteristics

The demographic and baseline characteristics of participants in the study, detailed in Table 1, demonstrate no statistically significant differences between the Low (*n* = 87) and High (*n* = 86) groups, including age, gender distribution, APACHE II scores, mNUTRIC scores, and baseline values for PA, RF-MLT, and RF-CS.

**Table 1:** Demographic and baseline characteristics of 173 participants

Variables	Low (*n* = 87)	High (*n* = 86)	*P*-Value
Age (year)	75 (65, 82)	76 (64, 83)	0.665
**Gender** (n, %)			1
Male	56 (64.37)	55 (63.95)	
Female	31 (35.63)	31 (36.05)	
**APACHE II score**	22 (18, 26)	23 (16, 26)	0.953
**mNUTRIC**	7 (6, 8)	7 (6, 7)	0.068
**PA**	80.93 (68.08, 93.62)	80.33 (67.57, 91.82)	0.794
**RF-MLT (**cm)	0.93 (0.84, 1.12)	3.85 (3.42, 4.28)	0.596
**RF-CSA (**cm^2^)	0.99(0.84, 1.13)	3.8 (3.47, 4.12)	0.419
**Weight** (kg)	66.02 ± 11.76	67.24 ± 8.19	0.429
**BMI** (kg/m^2^)	22 (20, 25)	22 (19, 24)	0.282
**Sepsis** (yes) (n, %)	15 (17.24)	20 (23.26)	0.472
**Reason for ICU admission** (n, %)			
Respiratory	41 (47.13)	37 (43.02)	0.697
Digestive	22 (25.29)	22 (25.58)	1
Urinary	12 (13.79)	10 (11.63)	0.842
Neurological,	11 (12.64)	16 (18.6)	0.384
Others	1 (1.15)	1 (1.16)	1

Notes: Continuous data were expressed as medians [interquartile ranges (IQR)] or means ± standard deviation (mean ± SD), and categorical data were presented as numbers (%). *P* < 0.05 was considered statistically significant between the Low and High groups

### Primary and secondary outcomes

**Fig. 2:**
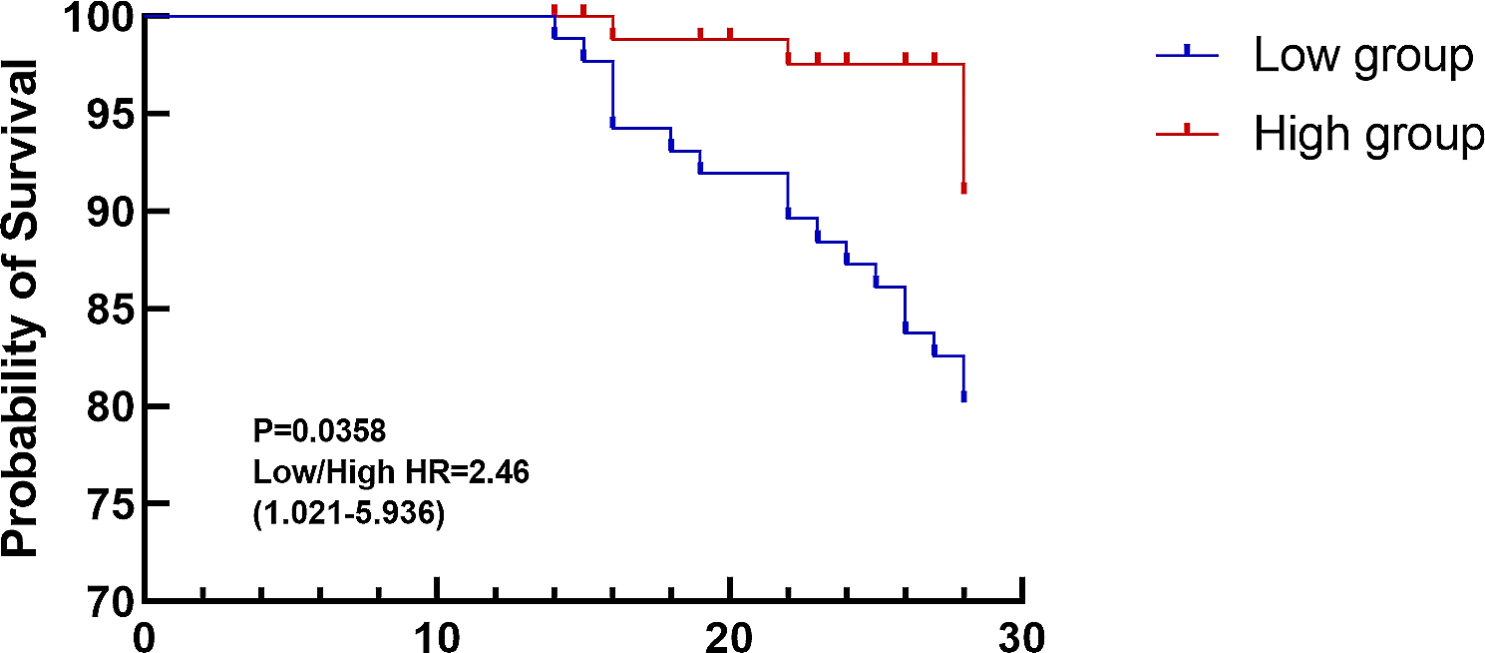
Survival curves of low and high groups

For the primary outcome of 28-day mortality, the rate was 19.54% in the Low group and 8.14% in the High group, but this difference was not statistically significant (*P* = 0.051). Regarding secondary outcomes, there were no differences between the two groups in the incidence of refeeding syndrome and the EN tolerance score. We further analyzed the 28-day mortality rate and the incidence of refeeding syndrome by incorporating the time factor using Cox proportional hazards models. The analysis showed that the Low group had a significantly higher 28-day mortality rate, with a hazard ratio (HR) of 2.462 (95% CI: 1.021 to 5.936, *P* = 0.045). In contrast, the risk of refeeding syndrome showed no significant difference between groups, with an HR of 0.784 (95% CI: 0.416 to 1.477, *P* = 0.452). Compared to the Low group, the High group exhibited a significantly reduced duration of ICU stay (*P* = 0.025). The comparison of mechanical ventilation duration between the Low group and High group yielded significance with a *P*-value of 0.039 (Table 2). Figure 2 illustrates the survival curves for both groups. The curves indicate a higher survival rate in the High group than Low group, although this difference did not reach statistical significance.

**Table 2:** Clinical outcomes of 173 participants

Variables	Low (*n* = 87)	High (*n* = 86)	*P*-Value	HR (95%CI)	*P*-Value
Mechanical ventilation duration (day)	10 (7.5, 13)	9 (7, 11)	**0.039**		
ICU stay (day)	12 (9, 15)	10 (8, 14)	**0.025**		
28-day mortality rate (n, %)	17 (19.54)	7 (8.14)	0.051	2.462 (1.021, 5.936)	**0.045**
Refeeding syndrome incidence rate (n, %)	17 (19.54)	22 (25.58)	0.442	0.784 (0.416, 1.477)	0.452
EN tolerance score	3 (1.5, 6)	3 (2, 7)	0.129		

Notes: Continuous data were expressed as medians (IQR). Categorical data were presented as numbers (%). *P* < 0.05 was considered statistically significant between the Low and High groups

Continuous measurements were conducted to assess important indicators of patient nutrition (PA) and kidney function (BUN/Crea). Serum levels of PA were measured on the 1th, 3rd, 5th, and 7th days after entering ICU for treatment. Serum BUN/Cr was measured on the 1th and 5th days. there was no significant difference in serum PA levels between the two groups. However, on the 7th day, the High group showed significantly higher PA level than the Low group. For BUN/Crea, there were no significant differences between the two groups (Table 3).

**Table 3:** Biochemical markers

Variables	Low (*n* = 87)	High (*n* = 86)	*P*-Value
PA (D_1_)	75.57 (62.2, 86.44)	75.20 (60.8, 86.54)	0.958
PA (D_3_)	138.79 (127.44, 150.2)	137.05 (126.87, 148.44)	0.465
PA (D_5_)	201.58 (125.92, 251.01)	183.55 (124.59, 247.53)	0.553
PA (D_7_)	144.54 (132.6, 153.74)	240.01 (172.72, 256.5)	**< 0.001**
BUN/Cr (D_1_)	17.57 ± 4.19	17.18 ± 4.59	0.567
BUN/Cr (D_5_)	17.53 ± 4.30	19.11 ± 5.64	**0.040**

Notes: D_1_, D_5_, and D_7_ mean the 1st,3rd, 5th, and 7th day treatment in the ICU/EICU, respectively. Continuous data were expressed as medians (IQR) or mean ± SD. PA, mg/L. *P* < 0.05 was considered statistically significant between Low and High groups

The RF-MLT and RF-CSA have important diagnostic value in assessing patient prognosis, and thus, we used ultrasonography for monitoring (Table 4). On the 5th day of treatment in the ICU, the atrophy rate of RF-MLT and RF-CSA in the High group was lower than that in the Low group, although this difference was not statistically significant (*P* > 0.05). On the day of ICU discharge, the atrophy rates of RF-MLT and RF-CSA in the High group were both significantly lower than Low group (*P* < 0.05).

**Table 4:** Atrophy rate of thickness and cross-sectional area of rectus femoris in subjects

Variables	Low (*n* = 87)	High (*n* = 86)	*P*-Value
RFMLT(D_5_-D_1_)/D_1_	15.43 (13.05, 19.22)	15.74 (11.63, 18.55)	0.380
RFCSA (D_5_-D_1_)/D_1_	15.06 (10.96, 17.65)	13.57 (11.24, 16.28)	0.080
RFMLT(_Dx_-D_1_)/D_1_	29.62 (23.35, 37.85)	27.90 (21.04, 31.45)	**0.002**
RFCSA (D_x_-D_1_)/D_1_	27.47 (121.84, 33.08)	20.46 (16.98, 23.46)	**< 0.001**

Notes: RF-MLT means rectus femoris muscle thickness, RF-CSA means rectus femoris cross-sectional area. D_1,_ D_5_ and Dx mean the 1st, 5th day treatment in the ICU/EICU, respectively. Continuous data were expressed as medians (IQR). *P* < 0.05 was considered statistically significant between the Low and High groups

In Tables 5 and 6, we demonstrate the impact of early high protein intake within subgroups of age (≤ 60 and > 60 years). In the stratification of > 60 years, the influence of high early protein intake on ICU stay time, PA levels, BUN/Crea, and the rectus femoris muscle atrophy rates remain significant. However, in ≤ 60 years stratification, only the effects on PA levels and the rectus femoris muscle atrophy rates are still significant. This discrepancy may be attributed to insufficient statistical power in the stratified analysis due to the sample size.

**Table 5:** Clinical outcomes (stratified by age, ≤ 60 years and > 60 years)

Variables	Low, ≤ 60 years (*n* = 12)	High, ≤ 60 years (*n* = 15)	*P*-Value	Low, > 60 years (*n* = 75)	High, > 60 years (*n* = 71)	*P*-Value	HR (95%CI)			
							≤ 60 years	*P*-Value	> 60 years	*P*-Value
Mechanical ventilation duration (day)	11.25 ± 3.84	9.80 ± 3.91	0.343	10 (7, 13)	8 (7, 10.5)	0.053				
ICU stay (day)	12.5 (9.75, 15)	14 (10, 15)	0.980	12 (9, 14.5)	10.87 (6.97, 14.78)	**0.013**				
28-day mortality rate (n, %)	3 (25)	2 (13.3)	0.632	14 (18.7)	5 (7.0)	0.066	1.922 (0.321, 11.524)	0.474	2.715 (0.978, 7.538)	0.055
Refeeding syndrome incidence rate (n, %)	2 (16.7)	5 (33.3)	0.401	15 (20)	17 (23.9)	0.707	0.529 (0.102, 2.731)	0.448	0.859 (0.429, 1.720)	0.668
EN tolerance score	3.67 ± 2.84	4.40 ± 3.92	0.579	3 (1.5, 5.5)	3 (2, 7)	0.132				

Notes: Continuous data were expressed as medians (IQR) or mean ± SD. Categorical data were presented as numbers (%). *P* < 0.05 was considered statistically significant between Low and High groups

**Table 6:** Biochemical index and rectus femoris atrophy rate (stratified by age, ≤ 60 years and > 60 years

Variables	Low, ≤ 60 years (*n* = 12)	High, ≤ 60 years (*n* = 15)	*P*-Value	Low, > 60 years (*n* = 75)	High, > 60 years (*n* = 71)	*P*-Value
PA (D_1_)	76.93 (65.77, 84.51)	74.55 (68.7, 87.69)	0.884	75.57 (60.28, 86.82)	75.57 (60.28, 86.3)	0.986
PA (D_3_)	146.69 (135.58, 152.6)	131.95 (127.64, 146.98)	0.093	137.35 (126.22, 149.44)	137.35 (125.61, 148.4)	0.865
PA (D_5_)	215.58 ± 48.63	183.25 ± 57.29	0.126	201.58 (123.74, 247.43)	181.66 (123.15, 254.9)	0.868
PA (D_7_)	147.04 (139.96, 153.72)	252.78 (230.77, 261.2)	**0.001**	144.54 (132.11, 153.12)	238.66 (169.74, 255.11)	**< 0.001**
BUN/Cr (D_1_)	16.7 (14.12, 22.25)	12.3 (10.8, 17.6)	0.071	17.57 ± 4.07	17.75 ± 4.31	0.796
BUN/Cr (D_5_)	17.77 ± 4.68	17.97 ± 4.19	0.906	17.49 ± 4.27	19.35 ± 5.90	**0.032**
RFMLT(D_5_-D_1_)/D_1_	15.25 ± 3.70	14.74 ± 4.19	0.741	15.83 ± 3.84	15.31 ± 3.91	0.418
FCSA (D_5_-D_1_)/D_1_	11.13 (8.65, 16.49)	12.44 (10.92, 16.27)	0.486	15.22 (11.18, 17.65)	13.62 (11.24, 16.22)	**0.034**
RFMLT(_Dx_-D_1_)/D_1_	29.93 ± 7.04	23.72 ± 6.44	**0.027**	29.62 (22.5, 38.08)	28.22 (23.44, 31.6)	**0.01**
RFCSA (D_x_-D_1_)/D_1_	28.65 ± 7.11	21.08 ± 3.32	**0.004**	27.22 (21.84, 32.67)	20.46 (16.79, 23.19)	**< 0.001**

Notes: Continuous data were expressed as medians (IQR) or mean ± SD. Categorical data were presented as numbers (%). *P* < 0.05 was considered statistically significant between Low and High groups

### Impact of protein intake of septic and non-septic patients

Due to the systemic inflammation in septic patients, which may lead to severe organ damage, there might be higher demands for nutritional support. Therefore, we conducted a subgroup analysis to explore the impact of increased early protein intake on septic patients (Table 7). The results indicated that high protein intake had no significant impact on mechanical ventilation duration, ICU stay, refeeding syndrome incidence rate, and EN tolerance score. However, compared to the Low group, the High group showed a significant decrease in the 28-day mortality rate (*P *= 0.018). However, a small sample size may not provide sufficient power to detect significant differences, thus serving as a preliminary finding that requires further investigation with a larger sample size to validate these observed results.

**Table 7:** Clinical outcomes of septic and non-septic patients

Variables	Non-septic (*n* = 138)			Septic (*n* = 35)		
	Low (*n* = 67)	High (*n* = 71)	*P*-Value	Low (*n* = 20)	High (*n* = 15)	*P*-Value
Mechanical ventilation duration (day)	10 (7, 14)	8 (6.5, 11)	0.023	9.75 ± 2.49	10.60 ± 4.85	0.542
ICU discharge (day)	12 (9.5, 15)	10 (8, 13)	0.004	11.35 ± 3.07	13.27 ± 5.73	0.253
28-day mortality rate (n, %)	7 (10.45)	6 (8.45)	0.913	10 (50.00)	1 (6.67)	**0.018**
Refeeding syndrome incidence rate (n, %)	14 (20.90)	17 (23.94)	0.822	3 (15.00)	5 (33.33)	0.383
EN tolerance score	3 (2, 5)	4 (2, 7)	0.145	2.5 (1, 6.25)	3 (1, 8.5)	0.637

Notes: Continuous data were expressed as medians (IQR). Categorical data were presented as numbers (%). *P* < 0.05 was considered statistically significant between Low and High groups

## Discussion

In this prospective randomized controlled trial involving critically ill patients admitted to the ICU/EICU for at least 7 days, we observed that increasing early protein intake to 1.5 g/kg/d did not significantly affect the primary clinical outcome, 28-day mortality rate, and secondary outcomes including refeeding syndrome incidence rate and EN tolerance score. However, Cox regression analysis showed that high protein intake significantly reduced 28-day mortality. Additionally, high protein intake appeared to enhance patients’ nutritional status and accelerate recovery. Notably, early high protein intake significantly reduced the 28-day mortality rate in septic patients. These findings indicate that early enteral supplementation of a higher dose of protein (1.5 g/kg/d) is safe and effective for critically ill patients.

Prior studies on the effects of early protein intake in critically ill patients have yielded inconsistent results. Hartwell et al. conducted a retrospective study involving 274 non-volitional critically injured adults and found that patients achieving protein targets within the first 4 days in the ICU had the lowest mean number of complications and operations [12]. In contrast, Lin et al. reported an association between early low (0.38 g/kg/d) or high (1.68 g/kg/d) protein intake and increased 28-day mortality in critically ill patients compared to moderate protein intake (0.8 g/kg/d) [13]. Our study revealed that increasing early protein intake did not exhibit a significant impact on the 28-day mortality rate, refeeding syndrome incidence rate, and enteral nutrition tolerance score. However, it was noteworthy that a decrease in mechanical ventilation duration and ICU stay was observed. This finding contrasts with some earlier studies that suggested a potential association between protein intake and clinical outcomes [13, 14]. The differences in patient populations, intervention protocols, and study designs may contribute to these disparate results. Interestingly, our study did reveal a positive impact on patient outcomes, as evidenced by the reduction in both mechanical ventilation duration and ICU stay. In the EFFORT Protein trial, the high-dose protein group received a protein supplementation of ≥ 2.2 g/kg per day, a dosage considerably higher than the recommended range of 1.2–2.0 g/kg. Subgroup analysis revealed negative impacts of this dosage on patients with kidney injury and organ failure [15, 16]. Consequently, while increasing early protein intake in critically ill patients may be beneficial, the optimal dosage and safe range remain to be clarified. Caution is particularly warranted in patients with kidney injury and organ failure.

In the management of critically ill patients, appropriate energy and protein intake is crucial for recovery and prognosis. The aim of our study was to investigate the effects of increased early protein intake on patient outcomes in a critical care setting. In our design, protein intake levels were intentionally varied between two groups, while non-protein calorie intake remained constant. Due to the caloric contribution of protein, this resulted in the High group receiving slightly higher total energy intake (an additional 2.8 kcal/kg/day), leading us to question whether the observed benefits were due to high protein or merely higher energy intake. To explore this issue, we reviewed some literature for established findings and compared them with our results. For example, a cohort study involving 113 ICU patients indicated that patients with lower provision of protein and amino acids had higher mortality rates. Cox regression analysis revealed that high protein provision significantly reduced the risk of death, even after adjusting for baseline prognostic variables such as APACHE II score, SOFA score, and age. However, no correlation was found between energy provision and mortality rates [17]. Moreover, studies have shown that critically ill patients maintained on similar protein intakes, experiencing either permissive underfeeding (40–60% of calculated caloric requirements) or standard enteral feeding (70–100%), had similar 90-day mortality rates, with no statistically significant differences in feeding intolerance or length of hospital stay [18]. This underscores that adequate protein intake appears more critical than caloric intake in improving the prognosis of critically ill patients. Thus, synthesizing these research findings, it seems reasonable to conclude that the benefits observed in our study can be attributed to high protein intake rather than the slight increase in total caloric intake. Nonetheless, nutritional interventions in critically ill patients are complex, and the contributions and demands of protein and energy to patient recovery still warrant further exploration to fully understand their interdependent effects.

Low serum ALB levels are associated with malnutrition and inflammatory states [19, 20]. In our study, we administer ALB to patients with hypoalbuminemia to maintain colloidal osmotic pressure, thereby preserving the body’s homeostasis. Furthermore, ALB has an average plasma half-life of approximately three weeks, exhibiting a relatively slow response to nutritional support [21]. Therefore, we did not use ALB as a nutritional indicator, but chose PA. In contrast to ALB, PA has a shorter half-life of about 2–3 days, offering a more immediate reflection of recent nutritional and metabolic changes [22]. Prior studies hinted at a potential rise in PA levels with early high protein intake in critically ill patients. However, inflammation, prevalent in such cases, could hinder PA synthesis, leading to static or reduced prealbumin levels [23]. Our study revealed a significant increase in PA levels in the High group compared to the Low group on the 7th day, indicating that increased early protein intake improves the nutritional status of critically ill patients. Furthermore, high protein intake may lead to nitrogen accumulation in the body, increasing urea production and potentially burdening the kidneys [23]. We did not observe a decline in kidney function in our study, suggesting that a dosage of 1.5 g/kg/d is relatively safe and effective. Nevertheless, personalized assessments should be conducted when devising protein support strategies to establish reasonable supplementation targets.

Among numerous indicators and scoring systems for determining nutritional risk, few are applicable to critically ill patients in the ICU [24]. ASPEN recommends using the mNUTRIC and Nutritional Risk Screening 2002 (NRS-2002) scoring systems to assess the nutritional status of critically ill patients, while ESPEN considers them not to be the gold standard for critically ill patients [6, 25]. Considering that malnutrition typically manifests as a decline in skeletal muscle mass and strength, recent studies have focused on exploring the relationship between nutritional status and muscle consumption [26, 27]. Ultrasonography holds diagnostic significance for ICU-acquired weakness (ICU-AW) by measuring RF-MLT and RF-CSA [28, 29]. A recent study suggested that ultrasonography measurement of rectus femoris and rectus abdominis thickness is a simple and reliable method for assessing nutritional risk in ICU patients [30]. Puthucheary et al. found a significant decrease in thickness of the rectus femoris and vastus intermedius muscles and RF-CSA in patients entering the ICU [31]. Our study aligned with Puthucheary’s findings, notably demonstrating significantly lower rates of reduction in RF-MLT and RF-CSA in the High group compared to the Low group. Therefore, we argue that early high protein intake can improve the nutritional status of critically ill patients. As an indicator of nutritional risk in critically ill patients, atrophy rates of RF-MLT and RF-CSA are more sensitive than PA level.

Septic patients experience systemic inflammation, leading to high protein breakdown and decreased synthesis, necessitating higher protein intake. However, systemic inflammation may induce multiple organ failure in septic patients, making it challenging to support the hepatic and renal burden associated with high protein intake [9]. Some studies suggest that one of the adverse effects of early high protein intake is the inhibition of autophagy, which plays a crucial role in sepsis by clearing damaged organelles, infection factors, and regulating immune responses [32, 33]. Weijs et al. analyzed the effects of early high protein intake on septic and non-septic patients, revealing a lower in-hospital mortality rate associated with early high protein intake in non-septic patients, while no significant change in mortality was observed in septic patients [34]. In our study, early high protein intake significantly reduced the 28-day mortality rate in septic patients. Although our sample size (*n* = 34) is relatively small and may affect result stability, these findings are still suggesting that an appropriate increase in early protein intake may be beneficial for patients with sepsis. Based on the limitation of sample size in the current study, it is proposed that a larger sample size is needed in the future in order to further confirm the results of this study.

Our study is a prospective randomized controlled trial, allowing better control of the research process, data collection, and minimizing biases that may occur in retrospective studies. However, several limitations should be objectively acknowledged. Our trial was conducted in a single-center, limiting the external validity of the results as patient characteristics may differ in other medical settings. The relatively small sample size, especially when differences between groups are not substantial, may restrict the statistical power of the study, making it challenging to detect some potential differences. Additionally, the 28-day observation period for clinical outcomes is relatively short, providing an incomplete assessment of the long-term effects of protein intake on patient outcomes. Despite some undeniable limitations, our study fills a specific gap in the existing literature by quantifying the immediate effects of protein intake on the short-term recovery of critically ill patients within a controlled environment. Additionally, we found that increasing early protein intake may also benefit critically ill patients with sepsis. In summary, this research lays a foundation for further large-scale studies, while expanding our understanding and application of nutritional science in critical care settings.

## Conclusions

Our findings reveal that while high protein intake did not directly reduce the 28-day mortality rate, it significantly enhanced recovery speed and nutritional status, evidenced by increased PA levels, reduced rectus femoris atrophy, decreased duration of mechanical ventilation, and shortened ICU stay. This study demonstrates the positive impact of early protein intake on the short-term recovery of critically ill patients, providing a scientific basis for further large-scale studies and informing clinical nutritional intervention strategies. These results underscore the importance of early nutritional support in critical care settings, suggesting that early high protein intake may play a crucial role in patient recovery and overall treatment success.

### Electronic supplementary material

Below is the link to the electronic supplementary material.

Supplementary Material 1

## Data Availability

No datasets were generated or analysed during the current study.

## Abbreviations

EN: Enteral nutrition
ICU: Intensive Care Unit
ASPEN: American Society for Parenteral and Enteral Nutrition
ESPEN: European Society for Clinical Nutrition and Metabolism
PN: Parenteral nutrition
EICU: Emergency ICU
mNUTRIC: Modified Nutrition Risk in Critically Ill
BMI: Body mass index
MAP: Mean arterial pressure
Lac: Lactate
NE: Norepinephrine
SPN: Supplemental parenteral nutrition
APACHE II: Acute Physiology and Chronic Health Evaluation II
ALB: Serum levels of albumin
PA: Prealbumin
BUN/Cr: Blood urea nitrogen/creatinine
RF-MLT: Rectus femoris muscle thickness
RF-CSA: Rectus femoris cross-sectional area
IQR: Interquartile ranges
D_1_, D_3_, D_5_: the 1st 3rd, and 5th day treatment in the ICU/EICU
Dx: the day of ICU discharge
ICU-AW: ICU-acquired weakness
NRS-2002: Nutritional Risk Screening 2002
