# Supplementary material for: Impact of early high protein intake in critically ill patients: a randomized controlled trial
Source: Nutr Metab (Lond). 2024 Jun 28;21:39. doi: 10.1186/s12986-024-00818-8 (PMC11212281; doi:10.1186/s12986-024-00818-8)
Supplement: Supplementary file 1 — Supplementary Material 1 [file 12986_2024_818_MOESM1_ESM.docx]

**Supplementary material**

Supplementary Table 1

Criteria for modified NUTRIC (mNUTRIC) score.

| Variables | Range | Score |
| --- | --- | --- |
| Year | <50 | 0 |
|  | 50-74 | 1 |
|  | ≥75 | 2 |
| APACHE II score | <15 | 0 |
|  | 15-19 | 1 |
|  | 20-27 | 2 |
|  | ≥28 | 3 |
| SOFA score | <6 | 0 |
|  | 6-9 | 1 |
|  | ≥10 | 2 |
| Number of comorbidities | 0-1 | 0 |
|  | ≥2 | 1 |
| Days in hospital before ICU | 0 | 0 |
|  | ≥1 | 1 |

Supplementary Table 2

Criteria for enteral nutrition tolerance scoring.

| Variables | Score | | | |
| --- | --- | --- | --- | --- |
|  | 0 | 1 | 2 | 5 |
| Bloating/Abdominal pain | NO | Mild/NO | Moderate (Intra-abdominal pressure 15-20 mmHg)/ Self-resolving | Severe (Intra-abdominal pressure > 20 mmHg)/ Not self-resolving |
| Nausea/Vomiting | NO | Yes/NO | Yes/Yes，250 mL < GRV < 500 mL | Yes/Yes，GRV > 500 mL |
| Diarrhea | NO | 3-5 times/d, < 500 mL | ≥ 5 times/d, 500 -1500 mL | ≥ 5 times/d, ≥ 1500 mL |
